# Smoking cessation attempts: is it useful to treat hard core smokers?

**DOI:** 10.1186/s12971-016-0100-0

**Published:** 2016-11-03

**Authors:** B. Joly, P. D’Athis, L. Gerbaud, J. Hazart, J. Perriot, C. Quantin

**Affiliations:** 1 Service de Biostatistique et d’Informatique Médicale (DIM), CHRU Dijon, Dijon, F-21000 France; 2 Service de santé Publique, CHU Clermont-Ferrand, EA PEPRADE4681 Université d’Auvergne, Clermont-Ferrand, France; 3 Dispensaire Emile Roux, Centre d’Aide à I’Arrêt du Tabagisme (IRAAT), Centre de Lutte Anti-Tuberculeuse (CLAT), 63100 Clermont-Ferrand, France; 4 INSERM, CIC 1432, Dijon, France; Dijon University Hospital, Clinical Investigation Center, Clinical epidemiology/clinical trials unit, Dijon, France; 5 Inserm UMR 1181 « Biostatistics, Biomathematics, Pharmacoepidemiology and Infectious Diseases » (B2PHI), Univ. Bourgogne Franche-Comté, F-21000 Dijon, France

**Keywords:** Cigarette consumption, Hard core, Quit attempt, Smoking cessation

## Abstract

**Background:**

Hard core smokers have been studied in many countries but only a few trials have compared the effectiveness of smoking cessation with other smokers. The objective of this study was to compare the frequencies of success in smoking cessation between hard-core smokers and other smokers.

**Methods:**

Data were collected in Clermont-Ferrand from the Emile Roux dispensary ‘Pneumology and Tobaccology Centre’ between 1999 and 2009. Assistance with smoking cessation was proposed to 1367 patients but only 1296 patients were included: 219 HCS and 1077 other smokers. Smoking cessation was considered a success when patients were abstinent 6 months after the beginning of cessation. The profiles of the two types of smokers were compared using Chi square test and Student’s t test. Multivariate logistic regression was used to investigate the association between the smoking cessation result and the type of smokers.

**Results:**

HCS more frequently consumed other psychoactive substances (41.1 % vs 25.7 % for other smokers; *p* < 0.001). Current depression was more frequent in HCS (46.6 % vs 34.8 % for other smokers; *p* = 0.001). Smoking cessation was less frequent in HCS (45.2 % vs 56.5 % for other smokers ; *p* = 0.002). In multivariate analysis, after controlling for other factors, the frequency of smoking cessation was not significantly associated with the type of smokers (*p* = 0.47). After limiting to initial factors (present before the beginning of smoking cessation), the frequency of smoking cessation was still not significantly associated with the type of smokers (*p* = 0.78).

**Conclusion:**

Smoking cessation is possible for hard core smokers, who should be treated as other types of smokers taking into account other factors:the problem is how to encourage them to try to stop smoking.

## Background

Smoking is the leading cause of avoidable death in the world [1]. According to the World Health Organisation smoking kills five million people every year, that is to say more than HIV, tuberculosis and malaria together [2]. In France, smoking is the leading cause of premature death (before 65 years of age).

Despite these figures, smokers still find it difficult to stop smoking: 73 % of smokers wish to stop; 22 % try and less than 5 % succeed without assistance [3]. It is suggested [4] that smokers who are more resistant to control smoking are less likely to quit. This hypothesis states that with the declining prevalence and growing social disapproval of smoking, smokers become increasingly socially disadvantaged [4, 5]. Hardcore smoking has become a major public health challenge for tobacco control and cancer prevention [1, 6–8]. So-called « Hard-core Smokers » (HCS) seem to be unaffected by prevention campaigns and tax increases on tobacco products: most of them have no intention, and never have had, to stop smoking because they love smoking and do not believe in its dangers [9–12]. Prevalence rates of hard core smoking are influenced by the criteria used to define the phenomenon and are affected by the lack of standard definition [13]. HCS smoke more than other smokers and therefore show more frequently comorbidities associated with smoking [14]. It therefore seems important to better understand the behaviour and profile of HCS to optimize the therapeutic strategy and the follow-up [9, 15]: HCS quickly begin to smoke regularly, have a greater level of consumption, and persist with their habit even though it makes them ill [10]. They frequently show higher levels of nicotine dependence [16–19]. They are more frequently men than women, and have lower levels of education and income, as they are often unemployed and live alone. They rarely have medical or dental cares. In addition, they do not admit their dependence on tobacco and frequently consume other legal or illegal addictive substances [20]. The proportion of HCS among smokers varies from 5 to 15 %, depending partly on the country [11, 20, 21].

The possibility of different strategies for smoking cessation in HCS has not yet been investigated, even though it is well-known that structured assistance gives smokers a greater chance of success [22]. The objective of the study was to compare the percentages of success in smoking cessation between hard-core smokers and other smokers.

## Methods

We included all persons attending in Clermont-Ferrand the Emile Roux dispensary ”Pneumology and Tobaccology Centre” where assistance with smoking cessation was proposed between 1^st^ January 1999 and 31^st^ December 2009 (1367 patients). These patients could be sent to the centre by their primary care physician or encouraged to go there by their families. Smoking cessation could also be initiated by personnel of the centre during a visit to the dispensary for another health problem. The use of the information collected for this study was declared to the National Commission for Data Protection (CNIL n° 1873761) and our study adhered to the tenets of the Declaration of Helsinki.

After exclusion for missing information about age, daily consumption of cigarettes or previous attempts to stop smoking, and for age less than 26 years, the cohort included 1296 patients (94.8 % of the attendants). A previous attempt to stop smoking was recorded (but the information about the date was not complete).

Finally, 219 patients were HCS (according to the most stringent definition in the literature [13, 20]: age 26 or over, more than 15 cigarettes a day and no previous attempt to stop smoking) and the remaining 1077 patients were other smokers.

The results of cessation were split into two categories (failure and success), according to the patient’s declarations and the measurement (6 months after the beginning smoking cessation) of level of carbon monoxide in exhaled air less of than 7 ppm.

The studied factors were:

- age, sex, number of pack years, consumption of another psychoactive product, number of previous cessation attempts, duration of the longest temporary cessation of smoking, former major depression, current depression, current anxiety, heart, lung or Ear-Nose-Throat (ENT) diseases involving smoking; Fagerström score, Richmond test, Demaria and Grimaldi test, position according to the Prochaska and Di Clemente cycle (Pre-contemplation, Contemplation, Preparation, Action and Maintenance);
- nicotine substitution, initial dose of nicotine in the replacement therapy, duration of the nicotine replacement therapy; treatment with serotonin reuptake inhibitors and duration of treatment; treatment with bupropion and duration of treatment; treatment with varenicline and duration of treatment (all durations counted in weeks);

daily consumption of cigarettes (when weaning began), weight gain (kilograms) from the beginning of withdrawal; perception of the assistance provided by the medical team during the follow-up, and perceived difficulty to stop smoking.Between-groups comparisons of distributions for categorical data (Richmond tests, Prochaska and DiClemente) were done with the Chi2 test, and Fisher’s exact test when there were not too many data. Comparisons of means (number of cigarettes; Fagerström, Demaria and Grimaldi scores; nicotine dose and durations; weight gain after treatment) were done with Student’s t test. Multivariate logistic regression was used to investigate the association between the smoking cessation result and the type of smokers. The first model included, all variables that were found by unifactorial analysis significantly associated (*p* < 0.10) with cessation, except for “previous attempts at smoking cessation” and “number of cigarettes per day” (because they were used to define the groups). The variable ‘age’ was introduced in the model as a binary variable with a cut off at 45 years (overall age median). The second model kept only the variables defined at the initial consultation which were found by unifactorial analysis to be significantly associated (*p* < 0.10) with cessation. The data were analysed using R 2.10.1 software.

## Results

A. Unifactorial analysis
  HCS were significantly less frequent than other smokers to stop smoking (Table 1). They were also more frequent to consume other psychoactive substances, and to show current depression.

**Table 1:** Proportions of the two sub-populations according to the different binary variables

Binary variables	Other smokers	HCS	*P*
Sex(proportion of men)	47.3 %	57.5 %	0,006
History of depression	34.4 %	34.7 %	0,92
Current depression	34.8	46.6	0,001
Current depression with history of depression	19.7 %	26 %	0,03
Current anxiety	36.7 %	42 %	0,14
Previous attempt	98.4 %	0 %	<0.001
Other psychoactive substance	25.7 %	41.1 %	<0.001
Cardiac diseases, pulmonary and ENT	50.6 %	55.3 %	0,21
Nicotine substitution	88.7 %	87.7 %	0,67
Smoking cessation Success	56.5 %	45.2 %	0,002
Treatment with serotonin reuptake inhibitors	50.7 %	51.6 %	0,81
Treatment with varenicline	5.5 %	7.7 %	0,18
Treatment with bupropion	14 %	9.6 %	0,08
Age (proportion of 45 years and under)	51.2 %	48.9 %	0.53
  The proportions for treatment with Varenicline (over 75), and the proportions for Bupropion (over 172), did not differ significantly between the two groups (Table 1). Weight gain after withdrawal was more frequent in other smokers than in HCS (*p* = 0.004) (Table 2).

**Table 2:** Means of the two sub-populations according to the different quantitative variables

Quantitative variables mean (SD)	Other smokers	HCS	*P*
Age	45.6 (10.6)	45.9 (11.1)	0.73
Fagerström score	7.2 (2.1)	8 (1.7)	<0.001
Daily consumption of cigarettes	24.2 (10.7)	28.1 (11.6)	<0.001
Number of pack years	29.7 (18)	33.9 (19.7)	0.002
Number of previous cessation attempts	2.8 (2.8)	0 (0)	<0.001
Demaria and Grimaldi test	11.7 (3.1)	11 (3.1)	0.003
Initial dose of nicotine in the replacement therapy	26.2 (12.1)	29.6 (14.1)	<0.001
Duration of the nicotine replacement therapy (weeks)	14.6 (15.6)	28.1 (17.5)	0.008
Duration of treatment with serotonin reuptake inhibitors (weeks)	6.7 (5.1)	7 (4.1)	0.7
Duration of treatment with Bupropion (weeks)	3.1 (1.2)	3.5 (1.6)	0.12
Duration of treatment with Varenicline (weeks)	5 (2)	5.3 (2)	0.6
Weight gain (kilograms)	4.1 (3.8)	3.9 (2.9)	0.52
  Means were significantly higher in HCS for daily number of cigarettes (*p* < 0.001) and cigarette pack-years (*p* = 0.002). The same was true for the Fagerström index (*p* < 0.001) which was higher in HCS.
  The Richmond results depended significantly on the group (*p* < 0.001): the high results (great motivation to stop smoking) were less frequent in HCS, who were therefore less motivated than other smokers to stop smoking. The differences for the Demaria and Grimaldi test were also significant (*p* = 0.003): scores were highest in the other smokers.
  The initial dose of the nicotine substitute (*p* < 0.001) and the duration of substitution (*p* = 0.008) were higher in HCS. The differences for the duration of treatment with bupropion and serotonin reuptake inhibitors were not significant.
  The perceived difficulty to stop smoking was significantly dependent on the group: it appeared greater for HCS (*p* = 0.03).The assistance provided by the medical team during the follow-up, as perceived by the patient was not significantly dependent on the group (*p* = 0.27) (but HCS appeared to be more sensitive than other smokers to the assistance).
  The status of the Prochaska and Di Clemente cycle depended significantly on the group (*p* < 0.001): more HCS (23.7 %) than other smokers (15.2 %) stay in the “pre-contemplation” level (decision to stop smoking).
B. Multifactorial analysis
  Multifactorial logistic regression analyses were carried out to look for an association between smoking group and smoking cessation, after adjustment for the other variables. For both models all variables were included in one step. The variables treatment with serotonin reuptake inhibitors, varenicline, or bupropion were excluded from the models due to convergence problems (too small number of patients taking these drugs). The type of smoker was not significantly associated with cessation of smoking whatever the model used.
  - Model with all variables significant in unifactorial analysis
    Most of the patients treated with varenicline or bupropion were not included in the model because their data ‘duration of nicotine substitution’ and ‘initial NS dose’ were missing.
    Consequently, there were 1049 patients (out of 1296) included in the analysis (Table 3).

**Table 3:** Model with all variables (present at the initial consultation and/or at follow-up) significantly linked with smoking cessation in unifactorial analysis (1049 patients)

	ORa	IC 95 %	*P*
Smoking group	0.84	0.53–1.35	0.47
Former depression	0.54	0.37–0.8	0.002
Current depression	1.06	0.7–1.62	0.78
Current anxiety	0.87	0.6–1.28	0.48
Other psychoactive substance	0.41	0.27–0.62	<0.001
Cardiac, pulmonary and ENT diseases	0.56	0.38–0.83	0.003
Richmond test			0.04
Demaria and Grimaldi test	1.03	0.97–1.09	0.35
Prochaska Cycle			0.01
Fagerström test	0.93	0.84–1.02	0.12
The assistance provided by the medical team during the follow-up, as perceived by the patient			<0.001
Perceived difficulty in smoking cessation			<0.001
Pack years	1	0.99–1.01	0.88
Initial dose for nicotine replacement therapy	0.98	0.96–1	0.07
Duration of nicotine replacement therapy	1.02	1.01–1.04	0.003
Weight gain (yes/no)	4.3	3.05–6.08	<0.001
Age (binary variable)	2.5	1.64–3.81	<0.001

All the variables present in the model are shown in this table

*ORa* adjusted OR
    The variable “smoking group” was not significant but smoking cessation (Table 3) was less frequent in patients a) who used other psychoactive substances, b) with heart-lung-ENT diseases and c) with former depression; it was more frequent in those with Prochaska cycle = 4 (smoker ready to change), and in those with assistance provided by the medical team during the follow-up, as perceived by the patient as very important. It was also more frequent in those with a longer duration of nicotine replacement. It was more frequent in those with weight gain after withdrawal and those with age over 45.
  - Model with only variables at inclusion found significant in unifactorial analysis
    Due to missing data, there were 1258 patients (out of 1296) included in the analysis (Table 4).

**Table 4:** Model with only variables at inclusion (present at the initial consultation) significantly linked with smoking cessation in unifactorial analysis (1258 patients)

	ORa	IC 95 %	*P*
Smoking group	0.94	0.66–1.33	0.72
Former depression	0.77	0.58–1.03	0.08
Current depression	0.92	0.67–1.27	0.63
Current anxiety	0.81	0.61–1.09	0.16
Other psychoactive substance	0.53	0.39–0.71	<0.001
Cardiac pulmonary and ENT diseases	0.64	0.47–0.86	0.003
Richmond test			<0.001
Prochaska cycle			<0.001
Fagerström test	0.92	0.86–0.99	0.03
Pack years	1.01	0.99–1.02	0.25
Demaria and Grimaldi test	1.07	1.02–1.12	0.004
Age (binary variable)	2.11	1.53–2.90	<0.001

All the variables present in the model are shown in this table
    The variable “smoking group” was not significant in this model either, but smoking cessation (Table 4) was less frequent in those with a) use of other psychoactive substances, b) heart, lung or ENT diseases and c) a higher score in the Fagerström test.
    It was more frequent in those with higher scores at the Richmond test, the Demaria-Grimaldi test and the Prochaska Cycle and those with age over 45

## Discussion

The unifactorial analysis showed that smoking cessation was less frequent in HCS (45 %) than in other smokers (56 %).

We think that the longer NRT in HCS may be explained by:

- weaning syndrome and/or craving are often intense; they are factors of smoking cessation failure in the short and medium term [9, 10].
- given this, the smoker received increased support, in the form of prolonged NRT associated with behavioral and cognitive support.

However, after adjustment for other factors impacting cessation, this result was not confirmed.

The benefits of smoking cessation are well known, especially with regard to cardiovascular and respiratory comorbidities [23, 24]but the presence of a disease was associated with lower frequency of smoking cessation. The desire to stop seems to be reduced when the disease is already present, which confirms reports by Scholte, Kotseva and Gregor [25–27].

It is well known that anxiety and depression are more frequent in smokers [28–30], and that quitting smoking is associated with a reduction in depression, anxiety, and stress, with an improvement in psychological quality of life and positive affects compared with continuing to smoke [31]. The associations between smoking cessation, anxiety and depression have been thoroughly studied by, for example, Taylor [32], Brown [33], McClave [34] and Zvolensky [35]. It is also known that former and current depressions have an impact on the risk of smoking relapse, when considered separately or together, and even after adjusting for anxiety disorders [36]. One interesting result of our study is that «former depression» stood out of “current depression” and “anxiety”, which was not intuitive because the disease was not necessarily present at the time of the study. Anxiety and current depression were no longer significant in multivariate analysis: this could be the result of an effective management of these diseases, either by the use of antidepressants, anxiolytics or other treatments.

In the model with all variables (i.e. at entry and during follow up) patients treated with varenicline or bupropion were not taken into account because nicotine replacement therapy (initial dosage and therapy duration) was not indicated for them. This is important because these treatments are now widely used. In contrast, the second model (i.e. at entry) includes all patients (with nicotine replacement therapy, varenicline or bupropion).

Finally, the principal criterion to distinguish between the two groups of smokers was their desire to stop smoking, as shown by the results of the Prochaska cycle and the Richmond test (which were worse for HCS) and, beyond that, their desire for access to care: by definition HCS are less motivated to stop smoking and another study showed that they were less likely to seek medical care [20].

After the other factors in the study were taken into account, there was no significant difference between the two groups of smokers for smoking cessation. We thus showed that hardcore smokers are able to quit. This result is coherent with previous results. Thus, Clare [5] showed that hardcore smoking declined among the high socioeconomic status smokers in Australia from 2001 to 2010., and Lund showed that the relative frequency of HCS in Norway declined in the period 1996–2009 from 30 to 23 % [37].

“The fact that clinicians, in our study, considered that HCS should require more intensive care may partially explain these results. We found that HCS required a higher initial dose of nicotine in the replacement therapy and longer nicotine replacement therapy. As a consequence, in the framework of a randomized double blind study, we might not have found the same results. However, we did not aim to conduct such a study; we wanted to know if, in clinical practice, taking into account the other main confounding issues, smoking cessation attempts would be useful. We think that our study also indicates that in an observational situation, where HCS seems to be attentively followed by clinicians, the results are promising.”

As the frequency of smoking cessation showed no great difference between HCS and other smokers, prevention campaigns should aim at promoting the cessation and access to care [14]. Moreover, among hard core smokers, distinct profiles based on perceived pros and cons of smoking exist [38]. Each profile might require a different tobacco control approach [38]. Further gains in smoking cessation must focus on understanding the characteristics of 'hard-to-engage' populations [39].

Finally, HCS differ from other smokers as they have never attempted to stop smoking, but once on the way to cessation their chances of success are satisfactory (45 % which is surprisingly good compared with 56 % among other smokers), which underlines the importance of treating HCS. Moreover, new strategies focused on HCS, like smoking reduction then cessation, appear effective to help hard core smokers to quit and reduce smoking [39] which is one more argument for encouraging HCS to try to quit smoking.

### Strengths and limitations

The strength of this study is to show that success is satisfactory for HCS engaged in smoking cessation. Besides, once other factors were taken into account, the frequency of cessation in HCS did not differ from that in other smokers: this is very important because it shows that HCS have a reasonable chance to stop smoking. They should therefore not be neglected by caregivers as they are not doomed to remain smokers all their lives. The question now is how to encourage them to begin a process of cessation. Our result is a clear indication that the other factors described in the study need to be taken into account in the management of HCS, in particular their motivation and the maturity of their decision to stop, even if it means delaying the start of cessation to obtain better motivation.

The main limitation of our study is that our sample of HCS may be biased as they all consulted at a smoking cessation center: our HCS could thus be more inclined to stop smoking than those in the general population. This possible bias is difficult to avoid if we want to study smoking cessation in HCS. Another limitation is that other definitions of HCS are possible such as “smoker aged 26 or older, with at least 15 cigarettes a day, no attempt to stop smoking in the previous year and uninterrupted smoking for 5 years” [40]. This definition would have made our HCS group larger and the other-smoker group smaller, and the differences between the two groups would have been smaller. Another limitation is related to the choice of the initial dose of nicotine in replacement therapy as the relevant variable. We chose to use the initial NRT dose as it was systematically recorded in the medical record. It would have been important to take into account the eventual dose of nicotine susbtitution, but this was not systematically available. In fact, this initial dose was very rarely modified.

Given these results, further research is needed to know how to encourage HCS to try to stop smoking. Other studies would be useful to compare HCS with Heavy Chronic Smokers, another kind of obstinate smokers, to identify the aspects that distinguish these two populations on smoking cessation prognosis.

## Conclusion

After adjustment for different factors, the chances of success for HCS were not significantly different from those for other smokers. This underlines the need to encourage HCS to make an attempt to stop smoking. Smoking cessation centres have a part to play with regard to HCS. The evolution of their motivation, or the maturation of their decision, plays a key role.

As the HCS status is probably acquired progressively, studies are needed to identify the pathways that lead a smoker to HCS status, and peculiarly sociological profiles. Better knowledge of HCS would thus make it possible to establish specific prevention strategies for this category of smokers. In the same way, once cessation has been achieved, the phenomena that push the obstinate smoker to smoke again need to be identified during follow-up so as to establish strategies to eliminate smoking relapse. Through prevention, it is essential to stop the emergence of HCS to reduce its frequency among smokers.

## Abbreviation

ENT: Ear-Nose-Throat
HCS: Hard-core smokers
